# Supplementary material for: Development and evaluation of ‘Sleep, Baby & You’—An approach to supporting parental well-being and responsive infant caregiving
Source: PLoS One. 2020 Aug 7;15(8):e0237240. doi: 10.1371/journal.pone.0237240 (PMC7413483; doi:10.1371/journal.pone.0237240)
Supplement: Attachment — Submitted filename: PLOSone_April 2020.docx [file pone.0237240.s001.docx]

*PLOS ONE* Review

April 22, 2020

ID: PONE-D-20-03162

**Title**: Development and evaluation of “Sleep, Baby & You” -- an approach to supporting parental well-being and responsive infant caregiving.

**Overall**: This study summarizes several stages of development of a family-level intervention to better manage infant sleep and parental well-being in early infancy. The study had four key strengths. First, it offers a novel, comprehensive intervention getting at both infant sleep but also management of parental mood symptoms (i.e., negative thinking and anxiety) via Acceptance and Commitment Therapy approaches. The inclusion of parental mood management strategies addresses an often-overlooked component of family functioning during early infancy. Second, the researchers were remarkably thorough in their developmental process of forming and evaluating this intervention. They first selected an intervention based on systematic review of already established interventions and then had contact with the authors of the selected intervention. They had thorough exposure to the selected intervention before adapting it for the UK population. Next, they had stakeholder meetings with practitioners and then commissioned a graphic artist to ensure that the intervention materials would be optimal for users. They then had providers and patients provide feedback via questionnaires, and they then field tested the intervention in five locations. This process was exemplary for intervention development, especially because the development incorporated both provider and patient perspectives. The third strength of this study was its thorough, well-organized literature review in the introduction, summarizing the need for this intervention and prior literature on similar interventions. The authors’ inclusion of Table 1 was particularly helpful. The fourth notable strength of this study was that they included a summary of the initial feedback they received from the feedback survey tool. This feedback will be useful for future research and clinical teams who develop and test family-level sleep interventions. Despite these four notable strengths, the study in its current form would benefit from a few improvements (mostly clarifying details), which are outlined below.

1. This phrasing in the abstract is slightly misleading: “Disrupted parental sleep, presenting as post-partum fatigue and as problematic infant sleep” because it implies that disrupted parental sleep presents as/manifests as problematic infant sleep when really the authors might mean that disrupted parental sleep may be caused by fragmented infant sleep or being disturbed by infant night awakenings. I suggest rephrasing/restructuring that in the abstract.
2. Also in the abstract, it would be helpful to know more about the number of participants. It is helpful to know that the intervention was field tested at 5 sites, but please report in the abstract how many parents and practitioners participated in the stakeholder meetings and how many families actually received the intervention in the five clinics.
3. Similarly, more clear description should be provided throughout the manuscript about the numbers of providers and patients, and it would be most helpful if this information were presented in a summary table in addition to in the text
   - There were six stakeholder discussion groups, each involving 3 to 15 participants – can you please specify how many total participants in these meetings?
   - After the graphic artist was commissioned to depict the key intervention messages, stakeholders reportedly shared drafts with colleagues – how many colleagues viewed the drafts?
   - “64 practitioners and parents” reportedly completed the feedback questionnaire – how many were practitioners and how many were parents?
   - I suggest moving the numbers about the trainings for phase 1 up to when the training is first discussed instead of in the evaluation of outcomes section.
   - 16 families consented to be contacted and 12 actually provided feedback out of how many total families who received the intervention?
4. How much time did the intervention take to administer to families? This information seems particularly relevant to report since the authors mention “Most respondents felt they only had enough time to discuss some of SBY information with parents (79%) compared to all of the information (29%).”
5. The authors mention that “Lack of clients specifically seeking help with infant sleep was the main reason for not following the preferred delivery format”.  -- how was this assessed? Did providers bring up sleep as a potential issue for all families encountered or was sleep only brought up if the families brought it up? Sometimes families may not recognize sleep as problematic, at least at older ages, so they may not even think to bring it up. Additionally, if the providers brought sleep up, did they say “is sleep a problem for your child?” or did they say “tell me about your child’s sleep” and then assess whether or not sleep was problematic?
6. The authors mention that treatment families implemented and sustained the suggested changes for 2 weeks. Could the authors add to the discussion section something about the impact of 2 weeks of changed behavior and/or mention as a limitation/direction for future research the need for additional follow ups later in time?
1. **Is the manuscript technically sound, and do the data support the conclusions?**

Yes, but it would be helpful if some data (i.e., numbers of participants and practitioners) were presented more clearly, such as in a summary table in addition to the written text.

2. **Has the statistical analysis been performed appropriately and rigorously?**

The statistical analysis required for this study was minimal as it was primarily about the development of an intervention and the initial responses to this intervention. Therefore, the included statistical analysis is sufficient.

3. **Have the authors made all data underlying the findings in their manuscript fully available?**

Yes, to the best of my knowledge.

4. **Is the manuscript presented in an intelligible fashion and written in standard English?... the language in submitted articles must be clear, correct, and unambiguous. Any typographical or grammatical errors should be corrected at revision, so please note any specific errors here.**

Yes, I found this manuscript to be easy to understand with little to no writing errors.
