# Supplementary material for: Development and evaluation of ‘Sleep, Baby & You’—An approach to supporting parental well-being and responsive infant caregiving
Source: PLoS One. 2020 Aug 7;15(8):e0237240. doi: 10.1371/journal.pone.0237240 (PMC7413483; doi:10.1371/journal.pone.0237240)
Supplement: Attachment — Submitted filename: Response to reviewers.docx [file pone.0237240.s002.docx]

Below please find our point by point responses to the reviewers’ comments in red text.

----------------

*PLOS ONE* Review

April 22, 2020

ID: PONE-D-20-03162

**Title**: Development and evaluation of “Sleep, Baby & You” -- an approach to supporting parental well-being and responsive infant caregiving.

**Overall**: This study summarizes several stages of development of a family-level intervention to better manage infant sleep and parental well-being in early infancy. The study had four key strengths. First, it offers a novel, comprehensive intervention getting at both infant sleep but also management of parental mood symptoms (i.e., negative thinking and anxiety) via Acceptance and Commitment Therapy approaches. The inclusion of parental mood management strategies addresses an often-overlooked component of family functioning during early infancy. Second, the researchers were remarkably thorough in their developmental process of forming and evaluating this intervention. They first selected an intervention based on systematic review of already established interventions and then had contact with the authors of the selected intervention. They had thorough exposure to the selected intervention before adapting it for the UK population. Next, they had stakeholder meetings with practitioners and then commissioned a graphic artist to ensure that the intervention materials would be optimal for users. They then had providers and patients provide feedback via questionnaires, and they then field tested the intervention in five locations. This process was exemplary for intervention development, especially because the development incorporated both provider and patient perspectives. The third strength of this study was its thorough, well-organized literature review in the introduction, summarizing the need for this intervention and prior literature on similar interventions. The authors’ inclusion of Table 1 was particularly helpful. The fourth notable strength of this study was that they included a summary of the initial feedback they received from the feedback survey tool. This feedback will be useful for future research and clinical teams who develop and test family-level sleep interventions.

Thank you for these positive comments. We have attempted to design and ‘pre-test’ this intervention as rigorously as possible so appreciate that this has been recognized.

Despite these four notable strengths, the study in its current form would benefit from a few improvements (mostly clarifying details), which are outlined below.

1. This phrasing in the abstract is slightly misleading: “Disrupted parental sleep, presenting as post-partum fatigue and as problematic infant sleep” because it implies that disrupted parental sleep presents as/manifests as problematic infant sleep when really the authors might mean that disrupted parental sleep may be caused by fragmented infant sleep or being disturbed by infant night awakenings. I suggest rephrasing/restructuring that in the abstract.

This has been rephrased. Our point is that for the most part ‘infant sleep problems’ are primarily problems for parents who are disturbed by infant night-waking that they perceive to be ‘infant sleep problems’ and consequentially infants being ‘treated’ in one way or another.

2. Also in the abstract, it would be helpful to know more about the number of participants. It is helpful to know that the intervention was field tested at 5 sites, but please report in the abstract how many parents and practitioners participated in the stakeholder meetings and how many families actually received the intervention in the five clinics.

We have included information in the abstract that 164 practitioners were trained in 5 different UK locations and that practitioners reported delivering the intervention (in full or in part) to at least 535 families (based on numbers reported by those practitioners who completed the feedback survey).

3. Similarly, more clear description should be provided throughout the manuscript about the numbers of providers and patients, and it would be most helpful if this information were presented in a summary table in addition to in the text

A summary table (Table 2) has been added as requested to page 10.

- There were six stakeholder discussion groups, each involving 3 to 15 participants – can you please specify how many total participants in these meetings?

Some participants attended multiple meetings. According to meeting attendance sheets there were a total of 26 different stakeholders who participated across all meetings. The breakdown is given in Table 2 on page 10.

- After the graphic artist was commissioned to depict the key intervention messages, stakeholders reportedly shared drafts with colleagues – how many colleagues viewed the drafts?

Stakeholders shared the drafts with 64 colleagues and parents who provided feedback as below. We do not know how many colleagues/parents viewed the drafts and did not provide feedback.

- “64 practitioners and parents” reportedly completed the feedback questionnaire – how many were practitioners and how many were parents?

24 identified themselves as parents, 40 as a health professional or other practitioner (e.g. baby massage). If they identified as both, we counted them as professionals. This information has been added to page 12.

- I suggest moving the numbers about the trainings for phase 1 up to when the training is first discussed instead of in the evaluation of outcomes section.

This has been moved to page 12 as suggested.

- 16 families consented to be contacted and 12 actually provided feedback out of how many total families who received the intervention?

The Health Visitors who participated in phase 1 of the evaluation did not consistently keep track of this information so we are unable to report this figure.

4. How much time did the intervention take to administer to families? This information seems particularly relevant to report since the authors mention “Most respondents felt they only had enough time to discuss some of SBY information with parents (79%) compared to all of the information (29%).”

Given the way UK Health Visitors work this is difficult to assess, as home visits cover a number of topics and discussions with parents do not always proceed in a linear manner. In different NHS regions we found HVs also had greater or smaller case-loads which meant there was variation in the amount of time an HV was able to spend with each family per visit. Some visits were 10 minutes long while others could last half an hour or more (especially if safeguarding issues were involved). We did not wish to add to the HV workload by asking them to keep track of the specific duration of any conversation where SBY was used. We have noted that this information is not available for us to report on page 15.

5. The authors mention that “Lack of clients specifically seeking help with infant sleep was the main reason for not following the preferred delivery format”.  -- how was this assessed? Did providers bring up sleep as a potential issue for all families encountered or was sleep only brought up if the families brought it up? Sometimes families may not recognize sleep as problematic, at least at older ages, so they may not even think to bring it up. Additionally, if the providers brought sleep up, did they say “is sleep a problem for your child?” or did they say “tell me about your child’s sleep” and then assess whether or not sleep was problematic?

This depended on individual practitioner preference as HVs have a large degree of autonomy in how they work. Some HVs only mentioned sleep when it was an issue raised by parents. Others incorporated enquiries about how parents were managing with sleep during routine contacts with all parents. This was an informal evaluation, not a rigorous research project, so we were unable to specify what HVs asked parents.

6. The authors mention that treatment families implemented and sustained the suggested changes for 2 weeks. Could the authors add to the discussion section something about the impact of 2 weeks of changed behavior and/or mention as a limitation/direction for future research the need for additional follow ups later in time?

Thank you – this is an important suggestion and has been added as a specific component to be addressed in future testing of this intervention on page 23.

1. **Is the manuscript technically sound, and do the data support the conclusions?**

Yes, but it would be helpful if some data (i.e., numbers of participants and practitioners) were presented more clearly, such as in a summary table in addition to the written text.

Added as suggested

2. **Has the statistical analysis been performed appropriately and rigorously?**

The statistical analysis required for this study was minimal as it was primarily about the development of an intervention and the initial responses to this intervention. Therefore, the included statistical analysis is sufficient.

3. **Have the authors made all data underlying the findings in their manuscript fully available?**

Yes, to the best of my knowledge.

4. **Is the manuscript presented in an intelligible fashion and written in standard English?... the language in submitted articles must be clear, correct, and unambiguous. Any typographical or grammatical errors should be corrected at revision, so please note any specific errors here.**

Yes, I found this manuscript to be easy to understand with little to no writing errors.

Thank you.

Reviewer #2:

Dr Douglas, the fourth author in this article, has recently circulated a petition to promote her Possums Sleep Programme approach to infant care in Australia. Supported by the views of parents and practitioners, this article proposes to import the Possums approach into the UK. While there is no doubting Dr Douglas’ commitment to this cause, a distinction has to be drawn between a mission and science. The goal of allowing parents to have choice is a worthy one, but there is a need to meet generic scientific and PLOS ONE criteria for balance, objectivity and safety. Unfortunately, the article as currently written does not meet these criteria. My aim below is to highlight the infant safety and other concerns involved. If the authors consider it is timely and wise to revise the manuscript, the individual points are designed to identify the concerns most in need of attention.

As this paper is about an adaptation of the Possums approach, which is not being led by Dr Douglas, and which has a different name from that used in Australia it is not clear to us why R2 begins their review of our paper with a comment about an Australian petition, which seems tangential as best. As R2 appears to be familiar with the petition mentioned we assume he must be aware that the petition commenced after both the completion of this evaluation and the submission of this paper. If the editors feel it is important to add Dr Douglas’s sharing of this petition on social media to the ‘Declarations’ section we will do so, however she was not the instigator of the petition which originated as a protest by a group of practitoners in favour of the Possums program to a funding award made by a state government to an organization with a very different philosophical approach. None of the UK authors were involved in this campaign and the UK team seeks to evaluate the principles and methods of the Possums approach under a renamed and adapted format in the UK which acknowledges Dr Douglas’s authorship of the Possums approach but is not led by her. We therefore refute the suggestion that this paper is the result of a ‘mission’ in the strongest possible terms. Indeed, as noted by R1 it reflects our efforts to document the development and evaluation of this intervention as transparently as possible prior to seeking RCT-funding.

We address R2’s comments regarding ‘balance, objectivity and safety’ as they appear below.

Abstract. ‘Previous research indicates that UK parents would value an approach that facilitates meeting their infants’ needs while supporting their own sleep-related well-being throughout their infant’s first year.’ As this wording makes clear, meeting infants’ needs is a key concern. The rest of this article must make clear where they are met.

As will become clear in the responses below, R2 and the authors of this article have very different understandings of the scope of ‘infant needs’. The authors of the submitted manuscript understand ‘meeting infant needs’ according to the principles of the Unicef UK Baby Friendly programme which is currently accrediting 92% of UK maternity hospitals and 91% of UK community services for parents and babies. This is encapsulated as: Supporting parents to respond to their baby’s needs for love, care and comfort in a way which promotes close parent-infant relationships and supports the mental health of both baby and mother (Unicef UK 2017: Guide to the Unicef UK Initiative Standards).

Abstract. ‘The intervention … helps parents support the development of their babies’ biological sleep regulators and promote their own well-being.’ This report does not provide any evidence that the Possums Sleep Programme helps to support the development of babies’ biological sleep regulators. The wording is misleading by implying the existence of this evidence, which is an exaggeration. There is some evidence that some parents value the Possums approach and the wording needs to be confined to this and other areas supported by evidence. The ‘Conclusions’ section of the Abstract is more accurate and balanced.

We can see how this statement needs clarification and have reworded to state that the results of this evaluation finds ‘The intervention … *offers parents strategies for* supporting the development of their babies’ biological sleep regulators’ (page 2). This is a factual statement about the content of the SBY discussion tool that was identified as being helpful by both practitioners and parents. Regarding the original Possums program, Dr Douglas and her Australian colleagues have published multiple peer reviewed papers detailing the evidence underpinning how the Possums approach supports the developing circadian cycle and the sleep-wake homeostat, and this approach can be reasonably described as ‘evidence-informed’. Until the approach is trialed on a large scale it will be impossible to capture and assess the evidence requested here by R2. This paper is a step towards amassing the practitioner and parent feedback needed to obtain funding for such a trial.

Competing Interests. Dr Douglas does declare an interest. However, I understand that she is financially supported by the Possums programme and may receive a salary from it. If so, it is normal practice in scientific journals to declare these interests.

No income is generated by Dr Douglas or any of the authors of this paper from the development of the Sleep, Baby & You intervention or the preliminary UK evaluation which is the subject of the manuscript under review. Dr Douglas already declares that she has a Medical Directorship role with the charity Possums Education – is R2 also asking her to declare how much she is remunerated for this role? This seems overly intrusive.

P5. ‘Treatment-oriented interventions often involve extinction techniques applied to infants who are causing parental sleep disruption (22,23,25–27). Examples of these approaches are summarised in Table 1’. These examples are biased, not representative. Two of them appear to have been chosen because of negative findings. It is true that such findings exist, but the majority of studies and systematic reviews show these methods to be effective in reducing infant night waking and crying out and enhancing infant sleep. The authors do themselves no favours by ignoring this evidence: see, e.g. Kempler et al. Sleep Medicine Reviews 29 (2016) 119e120. Also, Hiscock & Fisher Journal of Paediatrics and Child Health 51 (2015) 361–364. Table 1 needs to contain representative examples.

There is no claim made that Table 1 is ‘representative’—the table exists to support the point that ‘Treatment-oriented interventions often involve extinction techniques applied to infants who are causing parental sleep disruption’. The references cited support this point. The issue of effectiveness of these treatment options is a different point entirely which we do not raise here. Here we are demonstrating that approaches to resolving infant sleep issues may be treatment based (once issues have arisen) or preventative (to avoid issues arising) and we are providing indicative examples of each approach. The word ‘indicative’ has been added for clarity on page 5. The Kempler article is already cited in this paragraph (reference 14). The Hiscock and Fisher paper is not a primary research article regarding an intervention but an ‘annotation’ and is therefore not appropriate for citing in this paragraph.

P7. ‘The materials will be used as the basis for an intervention intended to reduce the proportion of parents experiencing postpartum depression, PPF and ISD and support to parents on meeting the needs of their infants…’ ISD is defined above as Infant-related Sleep Disruption. It needs to be made explicit that this involves parental sleep disruption and called IRSD, or PSD, since in the literature ISD is more commonly an abbreviation for Infant Sleep Disturbances (or Disorders).

We acknowledge our use of ISD may confuse some researchers and clinicians who are more familiar with this acronym as Infant Sleep Disorder and have amended Infant-related Sleep Disruption to IRSD throughout the manuscript.

The authors need to make it unambiguous throughout their article that they are not claiming to change infant sleep-waking behaviors – and that there is no evidence that Possums does so or meets the needs of infants.

In the submitted article we are only claiming to evaluate the Sleep, Baby and You intervention which is adapted from the Possums approach. ‘Meeting the needs of infants’ clearly means something different to R2 than it does to the authors. Sleep, Baby & You aims to encourage parents to ‘meet their babies’ needs’ by being responsive to their babies as per the current UK NHS and Unicef UK BFI guidance e.g. Unicef UK (2013) *The evidence and rationale for the Unicef UK Baby Friendly Initiative Standards*. R2 appears to use ‘meeting babies needs’ to refer to unspecified ‘sleep needs’ only. Sleep, Baby & You does not deny babies their sleep, and the program is not designed to treat babies with clinical sleep conditions.

P8. The Possums programme is also called ‘Neuroprotective Developmental Care (NDC) by its providers, but to the best of my knowledge there is no evidence that it protects neurological integrity or infant development. For this scientific report, there is no need for the phrase Neuroprotective Developmental Care and it would be better to omit it.

Here we are stating the current name of the Possums program for the purposes of transparency and clarity should readers wish to seek more information. A debate over the veracity of that name seems irrelevant here as this paper is not about the original Possums program, but the Sleep Baby and You adaptation. The Possums programs generally, of which the Possums Sleep Program is one domain, are referred to as Neuroprotective Developmental Care in both research publications detailing the evidence-base and in popular publications, and whilst R2 might not like this personally, it is not appropriate to delete this legitimate name.

P8. ‘The program educates parents in basic sleep science and how this relates to their infant’s sleep development. It offers strategies for optimising health function of the biological sleep regulators, to protect against excessive night-waking. It supports values-clarification and empowers parents to adjust their expectations and to experiment with how they might meet their babies’ needs. It supports cued care according to the most recent neuroscience and psychological attachment research, with the aim of promoting secure attachment, adequate sleep, and parent-infant enjoyment (34)‘ The reference given is to a small scale pilot study carried out by some authors of this article. That study did not provide any evidence that the Possums programme meets infant needs, or that it promotes adequate infant sleep or secure attachment. Here too the authors need to be unambiguous and evidence-based in their writing.

As noted above the authors of the Possums program have published multiple peer reviewed articles setting-out the scientific evidence upon which the program is based – we are therefore happy that this meets the criteria of an ‘evidence-informed’ program (and in fact were directed by a reviewer of the above-mentioned paper to use this terminology when referring to an as-yet-untested program). R2 objects to the statement on page 8 that the program educates parents about the topics listed. The statement made is accurate as the program **does** educate parents on these topics – but we have amended to state ‘is intended to educate’. Interested readers can easily access the Possums materials online to find out how parents are educated about these topics. Table 1 in the cited article (34) lays out the key principles of the Possums approach with 17 supporting references, making it an appropriate citation to underpin this program summary.

P 8-9. ‘The parent evaluation and Ball’s observations of the PSP in practice confirmed that the approach taken by this programme was consistent with a responsive approach and would be appropriate for development into a UK-based intervention.’. I think this is just saying that the first author approved it. In what way did scientific evidence confirm that the programme ‘would be appropriate for development into a UK-based intervention’? This sentence would be better omitted.

This sentence has been amended to make it clear the 1^st^ author was looking for a program for supporting parents with infant sleep that was a good fit with the responsive based models of parenting and infant-care currently being promoted as the ‘gold standard’ in UK NHS healthcare settings. The key criterion for selection was that it had to be compliant with Unicef UK BFI standards to promote the infant’s needs for contact, comfort and responsive parenting.

P9. ‘Stakeholders confirmed there was a gap in UK service provision addressing parental fatigue and sleep disruption during infancy.’ Yes, so far as this report is clear that Possums focuses on parental needs it is on firmer ground.

Sleep Baby & You focusses on parental needs, as this was the gap in provision identified by stakeholders. We are evaluating this adaptation, not the original program, and are not making a statement about the original Possums program.

P9. ‘As most community services in the region encouraged responsive parenting, extinction methods were considered inappropriate.’ Who considered them inappropriate? We need firm scientific evidence here. Most current health system and clinical sources I’m aware of recommend limited versions of what Hiscock and Fisher (see above) call infant behavioural management. Unless it can be supported, this claim should be omitted.

We are reporting here that the stakeholders who participated in the consultations considered extinction methods to be inappropriate. Perhaps R2 is not aware that the landscape of parent-infant facing NHS services in the UK has changed dramatically over the past decade, driven by the widespread desire of maternity, neonatal and community services for parents and infants to achieve Unicef UK Baby Friendly accreditation. As a consequence of their extensive BFI training for this accreditation a large number of health professionals working with mothers and babies consider extinction methods to be inappropriate for infants of any age.

Table 2. What does ‘dial baby down’ mean?

Taken from Possums workbook: parents offering cued care aim to keep the dial on the baby’s stress circuitry turned down as much as is sensibly possible.

P14. ‘Practitioners felt SBY also empowered and equipped … and they appreciated having a non-sleep training option to offer parents.’ Yes, supporting evidence-informed choice is desirable providing infant and parental health and safety are safeguarded. However, parents also need to be informed about the evidence for sleep training in an unbiased way. They can then choose according to their goals and priorities. It isn’t necessary to promote the Possums approach as the universal solution for all infants and parents.

This comment is misplaced. We are stating what practitioners told us in their feedback as we prepare to trial this intervention. We are not ‘promoting’ SBY as a universal solution, we are preparing to test it as an option to currently available interventions.

P 14. ‘I’m using it all the time, I think it’s amazing, there’s nothing that you teach that could do any harm so why wouldn’t you implement it?’ I appreciate that this is a participant comment, but it does get to the heart of the matter. Please see the final point below.

Is R2 suggesting we should censor the comments we received from HVs who provided feedback? See response to final point below.

P16. This needs to specify how many individual parents provided data and how many were mums or dads. The information above about families leaves this unclear.

The numbers are provided here and on pg13. We have added that only one parent responded per family (page 13). All parents who provided feedback by phone were mothers but we cannot confirm the role of those who provided feedback via the anonymous online evaluation form.

P17. ‘Improvements in frequent night waking and feeling stressed about their baby’s sleep were the most frequently reported positive outcomes.’ How many parents reported this? Please make clear: was the improvement in the parents’ night waking, their infant’s night waking behaviour, or the extent to which their infant’s behaviour was a problem for parents?

These details have been clarified on page 18.

P19. ‘Practitioners appreciated that SBY did not employ sleep training methods,’ how many – numbers and percentages are needed here.

Where specific numbers are available these have been added (page 14). On page 19 we have indicated that this statement is based on free text comments provided on the evaluation survey and comments made to the HB and CT via email which were not quantified.

P21. Discussion. ‘infant sleep disruption’ Having read this article through, I think this phrase is unhelpful and needs to be replaced. It is unclear whether the disruption involves infant or parental sleep and this ambiguity leads to a lack of clarity in the authors’ writing and potentially in readers’ understanding. ‘Infant-related Parent Sleep Disruption’ or simply Parent Sleep Disruption would be unambiguous. The phrase ‘Infant Sleep Disruption’ should be confined to measures of infants.

As previously noted this change has been made throughout the manuscript.

P23. ‘Although several trials have tested the effectiveness of educational interventions (based on sleep hygiene, self-settling, and the use of routines) alone or in combination with other psychological approaches (13,23–25) they have at best produced mixed results, and most have been found to be ineffective 1 (14,40–42) ‘Limit-setting’ educational approaches designed to support the development of ‘settled infant night-time behaviour’ have been examined as preventative interventions (7,24,26) but have been unsuccessful in achieving their primary outcome of increasing the duration of infant night time sleep.’ It is true that findings are mixed, but I believe this claim that most studies have found these methods to be ineffective is inaccurate. As noted above, I am not alone in that conclusion. More and better research is needed, but disregarding the published systematic reviews and meta-analyses is not scientific or helpful.

A statement has been added reiterating the point that researchers do not agree about the strength of existing studies and that better research is needed.

Overall, it is hard to disagree with the general point that many parents have unrealistic expectations for their baby’s sleep. The solution for that, though, is to provide accurate information. Although the evidence for the Possums approach is very limited, it appears that its primary benefits are for parents, particularly mums and particularly mums who can adapt to their baby’s sleep-waking habits. That is challenging to manage where both parents work office hours. There is firm evidence that infant crying which overwhelms parents can trigger shaken baby syndrome and other forms of infant abuse, raising safety concerns.

This comment does not address specific revisions for the manuscript under consideration. The point of the SBY intervention is to support parents in coping with babies’ typical sleep patterns. It will be trialed with parents from a wide cross section of the community including those who ‘work office hours’ as well as non-middle-class parents, non-white parents, and non-high income parents (e.g. broader than the typical pool of participants involved in behavior modification trials). It is not being offered to the general public in the UK prior to being rigorously tested via a trial that will capture both positive outcomes and adverse events.

Many parents and practitioners will have seen how distressed and wretched sleep-deprived babies become. Until Possums collects and provides measurements of infant sleep-waking and distress, the concern will remain that it helps infants less than the ‘infant sleep management methods’ referred to above. There is robust evidence from two genetic studies that the home environment – parenting not genes - makes the main contribution to individual differences in infant sleep-waking behaviour and problems (see e.g. Fisher et al.: www.pediatrics.org/cgi/doi/10.1542/peds.2011-1571). We know from the Gemini twin study that some infants need parental support to help them develop healthy eating habits. It would be a big surprise if that is not also true for infant sleep-waking behaviour.

This speculative comment does not address the submitted manuscript

A basic principle in both science and medicine is to do no harm. Archie Cochrane of Cochrane reviews fame developed that principle into the axiom that a new treatment should be introduced only when it is clear that it will do good and no more harm than existing treatments. My own view is that this article is premature. The priority is for the Possums approach to measure and publish evidence of any harms and benefits this approach has for infant sleep-waking and development more generally. There are simple parent-report methods for measuring infant sleep-waking and distress which, while not perfect, would provide evidence suitable for this purpose. It is puzzling that these authors have not used them. In the meantime, any findings published need to keep Cochrane’s principle in mind and to conform to scientific requirements for balance, objectivity and safety.

We’ve made clear in this paper that this evaluation of Sleep Baby & You (which is based on, but not identical to the original Possums program) is intended to inform future development and testing which will satisfy the need for evidence on the effectiveness of the program. R2 seems to be asking us to put the cart before the horse – one cannot (nowadays) trial an intervention without having demonstrated that there is a public need for it and that practitioners will use it. The useful point made by R2 here is that we need to make clear that no actual or anticipated harm was identified or reported by either practitioners or parents involved in the development or the evaluation phase, and this was something we asked them to do as part of the field-testing. This has been added to the end of the results section.
